# Consistent pattern of epidemic slowing across many geographies led to longer, flatter initial waves of the COVID-19 pandemic

**DOI:** 10.1371/journal.pcbi.1010375

**Published:** 2022-08-15

**Authors:** Michal Ben-Nun, Pete Riley, James Turtle, Steven Riley

**Affiliations:** 1 Predictive Science Inc., San Diego California United States of America; 2 MRC Centre for Outbreak Analysis and Modelling, Department of Infectious Disease Epidemiology, School of Public Health, Imperial College London, London, United Kingdom; Washington State University, UNITED STATES

## Abstract

To define appropriate planning scenarios for future pandemics of respiratory pathogens, it is important to understand the initial transmission dynamics of COVID-19 during 2020. Here, we fit an age-stratified compartmental model with a flexible underlying transmission term to daily COVID-19 death data from states in the contiguous U.S. and to national and sub-national data from around the world. The daily death data of the first months of the COVID-19 pandemic was qualitatively categorized into one of four main profile types: “spring single-peak”, “summer single-peak”, “spring/summer two-peak” and “broad with shoulder”. We estimated a reproduction number *R* as a function of calendar time *t*_*c*_ and as a function of time since the first death reported in that population (local pandemic time, *t*_*p*_). Contrary to the diversity of categories and range of magnitudes in death incidence profiles, the *R*(*t*_*p*_) profiles were much more homogeneous. We found that in both the contiguous U.S. and globally, the initial value of both *R*(*t*_*c*_) and *R*(*t*_*p*_) was substantial: at or above two. However, during the early months, pandemic time *R*(*t*_*p*_) decreased exponentially to a value that hovered around one. This decrease was accompanied by a reduction in the variance of *R*(*t*_*p*_). For calendar time *R*(*t*_*c*_), the decrease in magnitude was slower and non-exponential, with a smaller reduction in variance. Intriguingly, similar trends of exponential decrease and reduced variance were not observed in raw death data. Our findings suggest that the combination of specific government responses and spontaneous changes in behaviour ensured that transmissibility dropped, rather than remaining constant, during the initial phases of a pandemic. Future pandemic planning scenarios should include models that assume similar decreases in transmissibility, which lead to longer epidemics with lower peaks when compared with models based on constant transmissibility.

## Author summary

In planning for a future novel respiratory pandemic, or the next variant of SARS-Cov-2, it is important to characterize and understand the observed epidemic patterns during the first months of the COVID-19 outbreak. Here, we describe COVID-19 epidemic patterns observed in the U.S. and globally in terms of patterns of the time-dependent reproduction number, *R*(*t*), using an age-stratified compartmental model. We found that daily death data of the first months of the COVID-19 pandemic can be qualitatively classified into one of four profile types: “spring single-peak”, “summer single-peak”, “spring/summer two-peak” and “broad with shoulder”. Using the concept of local pandemic time, *t*_*p*_, we show a consistent pattern on four continents of an initial large magnitude and variance in reproductive number *R*(*t*_*p*_) that decreases monotonically and hovers around one for many days, regardless of specific intervention measures imposed by local authorities and without an accompanying decrease in daily death prevalence. We attribute this to significant behavior changes in populations in response to the perceived risk of COVID-19.

## Introduction

The roll out of effective vaccines [1, 2] and the emergence of more transmissible [3–5] and antigenically distinct [6] lineages of SARS-Cov-2 virus [7] marked the end of the global first wave of the COVID-19 pandemic. Over the first year, the COVID-19 pandemic has negatively impacted the health and well being of almost every population around the world. In the absence of an effective vaccine, most countries implemented non-pharmaceutical interventions (NPIs), including travel restrictions, school and work closures, social distancing, contact tracing, quarantining and mask requirements [8–10]. However, the extent of these measures, the degree of compliance with them, and their effectiveness, varied greatly from one setting to another and, even now, is not fully understood [11–15].

The transmission of the SARS-Cov-2 virus is often quantified using the time-varying reproduction number, *R*(*t*), which represents the mean number of secondary cases that a single primary case will infect. Many studies have focused on estimating the impact of different interventions on *R*(*t*) under the implicit assumption that interventions are similar between different populations [16–27]. However, the analytical approach in these studies conditions on the assumption that the interventions as measured are the main drivers of changes in *R*(*t*), with transmissibility assumed to be constant otherwise. The work presented here is different. We study overall trends in *R*(*t*) and do not explicitly consider individual interventions as explanations for a reduced value of the time-dependent transmission.

In this study, we compared daily mortality profiles at many locations by first qualitatively describing the data and categorizing it into four archetypes. For the quantitative analysis, we developed an age-stratified compartmental model [26] that fits a flexible, smoothly varying reproduction number and used it to study the epidemic, from January to October of 2020, in 49 jurisdictions in the contiguous U.S. and 89 locations globally. Our model allows for multiple values of *R*(*t*) and is an extension of our previous work which used a smoothly varying two-value functional form [28–30]. Although population disease profiles differ significantly temporally, we explored the idea that there may be similarities if studied from a common start time. We call this “local pandemic time” (*t*_*p*_, defined as the time elapsed since the first reported death). Trends in pandemic time *R*(*t*_*p*_) were compared to those of calendar time *R*(*t*_*c*_). Unlike earlier studies, our model used the inferred daily death as the “gold standard” data and we applied a Markov Chain Monte Carlo (MCMC) procedure [31] to fit *R*(*t*) to an increasing number of pandemic days. For each fitting time-window, we analyzed the value of *R*(*t*) for the prior two weeks and we discuss the results for the U.S. and globally without attempting to correlate any changes in *R*(*t*_*p*_) with specific NPIs.

## Methods

We describe methods in three parts: data, descriptive analysis, and models and fits. Additional details can be found in supplementary section Appendix A in S1 Text.

### Data selection

Many data streams can be used as a measure for the spread of COVID-19 [11–13]; however, daily confirmed number of cases, confirmed hospitalizations [1] and cumulative deaths are the most commonly used, and, in particular, these datasets as reported by the Johns Hopkins University (JHU) team [32]. Because of likely biases in the confirmed number of cases (e.g., the large change in test availability over time and changes in healthcare providers testing recommendations) and the limited availability of hospitalization data, we used the reported confirmed deaths as the most accurate and least biased measure of the pandemic. We note that the death data are also imperfect and may underestimate the true toll of the pandemic (Viglione et al. [33] and Centers for Disease Control and Prevention [34]). We started with the cumulative reported deaths as published by JHU [32] and inferred daily deaths for each location. Data for the study were retrieved from the JHU database in November, 2020. Irregularities in reporting occasionally resulted in negative incidence values for some days and these days were given a weight of zero in our fitting procedure. All other days with non-negative values were given an equal weight of one.

The data were split into two groups: U.S. and global. The U.S. dataset included all 48 contiguous states and the District of Columbia. Global data locations were chosen from both country level and administrative level-one divisions (state/province/territory/etc). Sub-country locations included those that appeared in the JHU database on April 31, 2020 (Canada, Australia), but excluded European island territories. Locations in the United States and China were also excluded from the global dataset. After these exclusions, the final list of locations in the global dataset was chosen as the top 110 by cumulative deaths. The 110 locations were grouped by continent (Africa, Americas, Asia-Oceania, Europe) for the analysis. Whereas all 110 locations were included in the descriptive analysis, only a subset of 89 locations with two or more weeks of daily death data by April 15, 2020 were included in the quantitative study.

Country level population totals and age distributions were taken from the United Nations World Population Prospects 2019 [35]. Male and Female populations were combined and five-year age bins were aggregated to the following 10-year bins: 0–9, 10–19, …, 70–79, 80+. For the United States, state-level populations and age distributions were taken from census data [36, 37], and converted to the same combined-sex and decadal ages format.

### Descriptive analysis

We analysed the inferred daily death data, for both the U.S. and globally, and qualitatively categorized it into one of four main profile types: “spring single-peak”, “summer single-peak”, “spring/summer two-peak” and “broad with shoulder”. Time series, for both the U.S. and the world were subjectively placed in the categories. While we used the northern-hemisphere seasons to label these profile types, we did not investigate the role of climatic effects which are beyond the scope of this study.

### Model and fits

A sketch of the model is shown in Fig A in S1 Text and it is described in detail in Appendix A in S1 Text. Here, we only highlight the main features of the model and the fits. We used an age-stratified SE[I]_4_RX compartmental model (where X refers to death and four levels of severity for the infectious compartments were included: asymptomatic, flu-like, mild and severe) to fit the inferred daily reported death for each location separately. (When mobility between countries/states is drastically reduced, as has been the case during the first nine months of the pandemic, it is appropriate to assume that while the date the virus is introduced to a community is determined by travel, the ensuing dynamics is often dominated by community transmission). Key parameters in the model are the age-specific probabilities of entering the severe infectious compartment, which can lead to death. These were taken from [38]. The age and country specific contact matrix was derived directly from the work of Walker et al. [39] using the“squire” R package available on github: https://github.com/mrc-ide/squire.

In previous studies on influenza and influenza-like-illness [28–30] we used a smoothly varying two-value functional form to describe the time-dependent reproduction number: *R*(*t*) = *β*(*t*)*γ*, where *β*(*t*) is the time-dependent transmission rate and *γ* is the total recovery rate. Here we extended this model to an arbitrary number of values:

$$\begin{array}{c} R\left(t\right)=\frac{1}{2}[R_{0}+R_{N}+\sum_{n=1}^{N}((R_{n}-R_{n-1})\text{tanh}(\frac{t-t_{n-1}}{L}))] \end{array}$$ (1)

This produces a smooth curve where at roughly time *t*_*n*−1_, the value of *R*(*t*) transitions from *R*_*n*−1_ to *R*_*n*_ with an approximate transition time of ≈ 2*L* days.

For each location, we determined the joint posterior distribution for the model parameters by fitting the inferred daily reported death using an adaptive step size MCMC [31] procedure with 10^6^ steps. Only the parameters that govern the time variation of *R*(*t*) were optimized (*R*_0_, …, *R*_*N*_ and *t*_1_, …, *t*_*N*−1_) and the timescale of variation was set to approximately seven days (using *L* = 3 in Eq 1). The objective function in the fitting procedure was a Poisson-based Log-Likelihood, and the fitting maximized the probability that the inferred daily reported death is a Poisson expression of the model daily incidence death. Multiple models of *R*(*t*) (with 2,3, 4 and 5 values, i.e. *N* = 1, …, 4) were fit to each location and the *AIC*_*c*_ [40] score was calculated for each model. We selected the best *N* based on *AIC*_*c*_ score (provided the effective chain size of all the parameters was greater than 50).

We used a simulate-and-recover procedure to validate the model. A known synthetic profile for *R*(*t*) was used to generate synthetic daily death incidence data. The model was fit to synthetic data and the recovered incidence and *R*(*t*) profile were compared with the known input (Fig B in S1 Text). The model was able to recover a large variety of synthetic profiles with high accuracy.

To investigate how *R*(*t*) evolved during the course of the pandemic we introduced the concept of “local pandemic time” (*t*_*p*_) defined as the number of days elapsed since the first reported death in a location. For each location, we simulated a retrospective study by repeating the fitting procedure using an increasing number of local pandemic days: we started with 30 days of data since the first reported death and increased it to 45, 60, 75, 90 and 105. We also fitted the reproduction number as a function of calendar (i.e. regular) time *R*(*t*_*c*_). Starting with data only until mid-April 2020 and increasing it in five increments of 15 days. Additionally, we calculated an apparent reproduction number, *R*(*t*) as it appeared at the end of each fitting period. For both the calendar and pandemic time calculations, the apparent reproduction number was calculated as the average value for the last two weeks of each study period. This analysis was first applied to the contiguous U.S. (48 states and the District of Columbia) and then extended to 89 locations outside the U.S. The characteristics of the daily inferred death profiles, of calendar and pandemic times *R*(*t*) and the similarities between the U.S. and the world are highlighted in the Results and Discussion sections.

## Results

**Fig 1:**
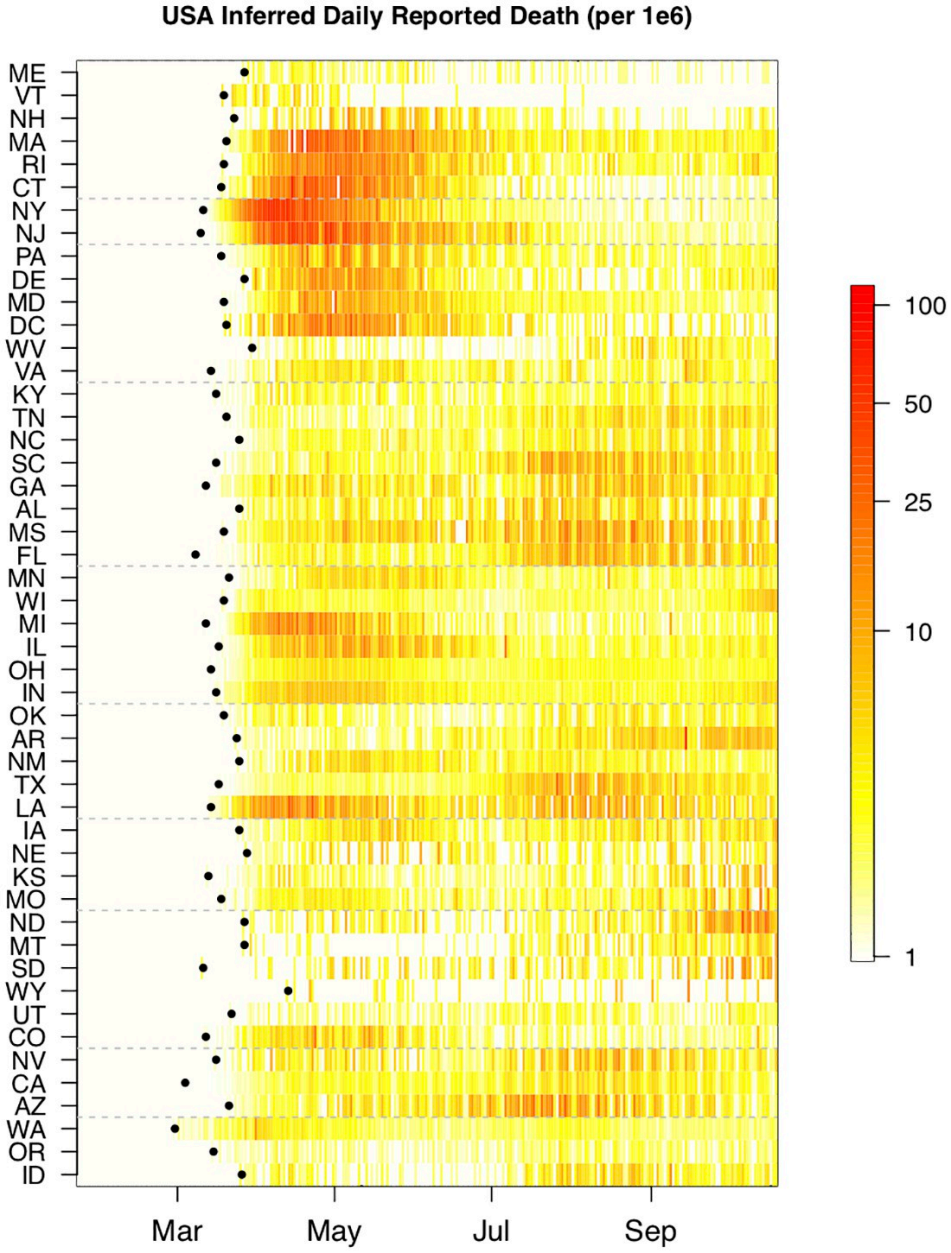
Inferred daily reported deaths (plus one) for the contiguous U.S. From top to bottom, the 48 states and the District of Columbia are ordered by the Human and Health Services (HHS) regions and within each region they are ordered by decreasing latitude. Regions are separated by a dashed grey line. For each location, the date of first reported death is marked with a black dot. Only the first nine months of the pandemic are shown. For clarity, the data are plotted on a log scale and normalized per 10^6^ people.

The U.S. outbreak was first detected in the state of Washington in late February [41]. The next six months of the pandemic can be qualitatively described as a sequence of four state-level archetypal epidemic curves of reported deaths (Fig 1). (As noted earlier, our choice of names for these four groups is descriptive only and does not imply any seasonal driving effects which are not within the scope of this study). The first shape appears as a “spring single-peak”. This north-east wave spread from New York and New Jersey to neighboring states (e.g., Connecticut and Massachusetts), and to the entire north-east corridor (e.g., Rhode Island, New Hampshire, Virginia, the District of Columbia, Maryland, Delaware and Pennsylvania). Overall, during this period, most states in HHS regions 1–3 exhibit the north-east profile with a large peak in daily deaths. We describe the second typical shape we observed as a “summer single-peak”. It is observed in a few states that avoided a spring peak but saw their first peak in the summer (e.g. South Carolina, Tennessee, Florida, Texas, Arizona, Arkansas and Idaho). The third typical shape exhibits two peaks, in both the spring and the summer. For example Georgia, Louisiana and Nevada. The fourth typical shape, “broad with shoulder”, comes from states that do not exhibit a clear sharp peak (e.g North Carolina, Kentucky, Oregon, Utah, and California) but rather a broad long shoulder-like profile with increases in deaths appearing only in the summer/early fall. Finally, we note that sparsely populated states (such as North and South Dakota), which largely avoided the spring and summer peaks, did not start to show increases in deaths until September.

**Fig 2:**
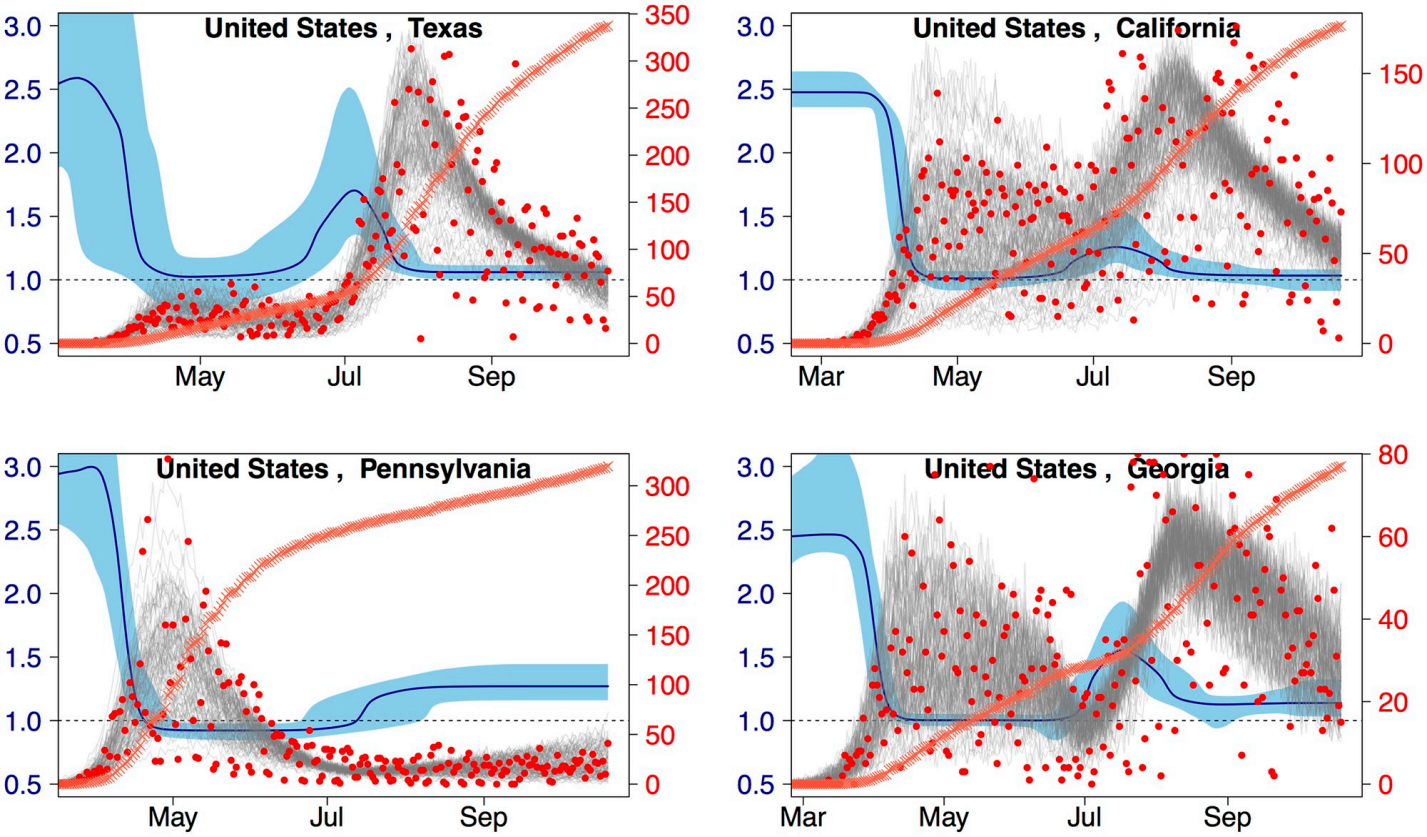
Fitting inferred daily reported deaths. Sample fits to inferred daily reported deaths (plus one) for four U.S jurisdictions red circles and right y-axis. The grey traces are 100 samples from the posterior distribution of the fit and the orange crosses denote the reported per capita cumulative deaths (no y-axis). The median and 95% confidence interval for *R*(*t*) is shown in dark and light blue with the left y-axis. Locations are ordered by decreasing cumulative deaths.

Individual model results appear in Fig 2 for four U.S. jurisdictions and in Fig E in S1 Text for 15 global locations. The four selected U.S. locations represent the different data profiles described above (summer peak, extended shoulder with summer peak, spring peak, and spring and summer peaks). Similarly, the global locations were chosen to be representative of four continents and all data profiles. The different epidemic profiles showed different patterns in the time-varying transmissibility when viewed in calendar time. For example, the profile for Pennsylvania started at *R* ≈ 3 before dropping to just below 1 in May. It maintained that value up to July before rising to ≈1.27. In contrast, Texas showed an initially high value (> 2.5), then dropped rapidly to values just above 1, until late June, at which point it increased to slightly more than 1.5 before returning to values hovering just above 1 for the remainder of the interval. The difference in complexity between the two inferred epidemic profiles was evident in the version of the model with the most parsimonious number of changepoints (see Methods). The number of changepoints with the lowest *AIC*_*C*_ for Pennsylvania was three whereas for Texas it was four. For California and Georgia the number of changepoints was also four. Whereas the details of the patterns in the time-varying transmissibility may vary between locations, we did find that for all four U.S. jurisdictions and most of the 15 locations (exhibiting very different dynamics) the initial value of *R*(*t*) was high (above 3 in Italy, Iran and Sweden) and it decreased to either below or around 1 (dashed grey line). In all cases the model produced a smooth result for *R*(*t*), and with its’ flexible form we were able to fit a variety of profiles from all four archetypes. The quality of the fits shown in Fig 2 and Fig E in S1 Text is representative of the results we obtained for all locations included in this study.

**Fig 3:**
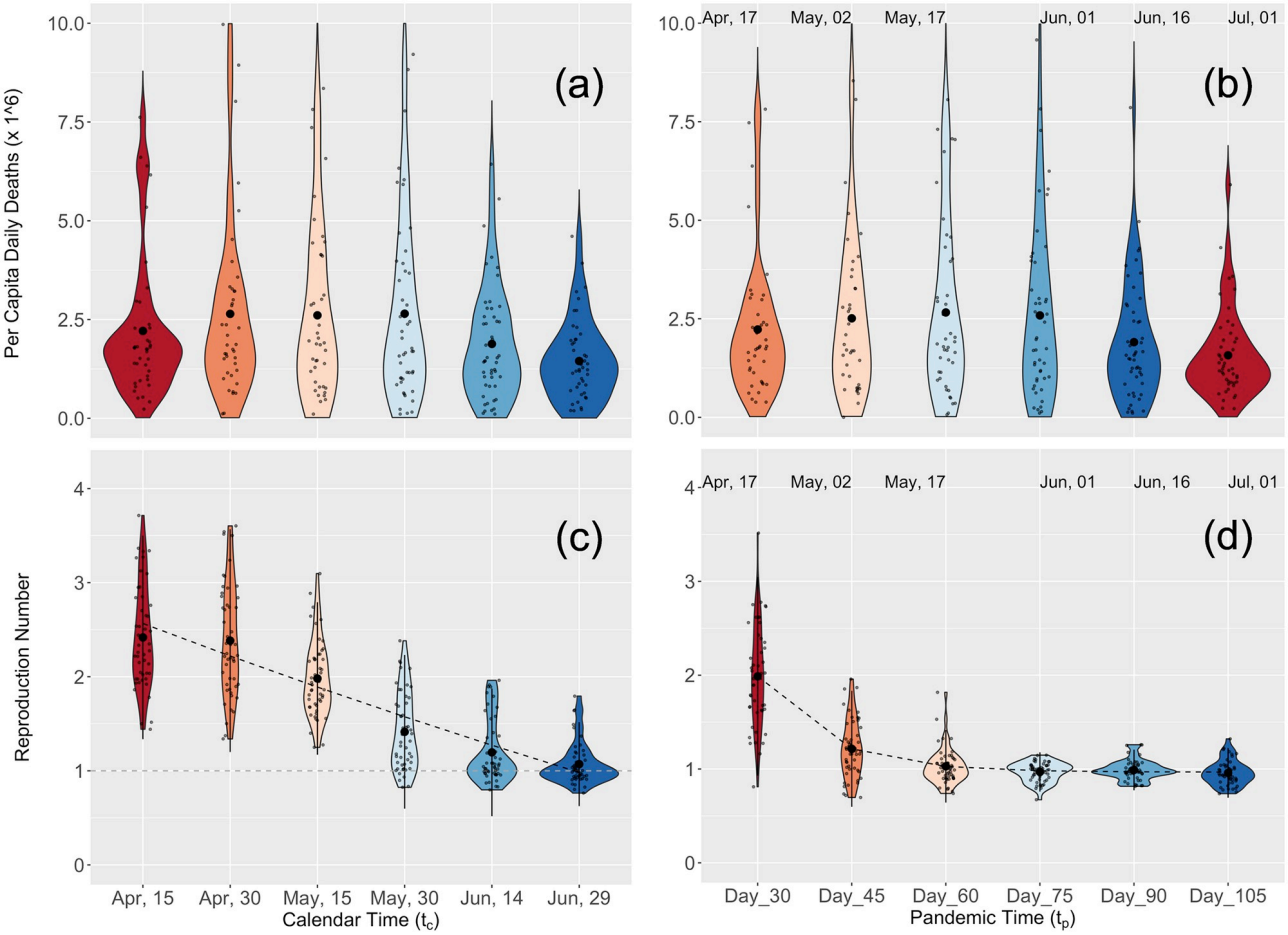
Calendar and pandemic time analysis for the U.S. A violin plot representation of per capita daily deaths for the contiguous U.S. and the distribution of calendar and pandemic times R values for the contiguous U.S. (top and bottom panels respectively). (a) Calendar daily death prevalence calculated using a (centralized) moving average of two weeks using the same dates as in panel (c). (b) Pandemic time daily death prevalence calculated for each jurisdiction using a (centralized) moving average of two weeks around the local pandemic date. (c) Calendar time R(*t*_*c*_) estimated using data from the first reported death in each jurisdiction up-to the date indicated in the panel and averaging the result for the last two weeks. (d) Pandemic time R(*t*_*p*_) estimated by fitting the first: 30, 45, 60, 75, 90, and 105 days after the first reported death in each jurisdiction and averaging the result of the last two weeks. The dashed black line in panels (c) and (d) shows an exponential fit to the results (see also Tables D and E of S1 Text). In panels (b) and (d), the mean date associated with the local pandemic times is indicated above each set of results. In all four panels, to increase readability, a jitter is applied to the displayed data points.

The distribution of deaths per capita across states in the continental U.S. was stable for the first half of the study. In contrast, the distribution of *R* numbers declined substantially during the same period (Fig 3). To simulate a retrospective study, we refitted overlapping subsets of the daily death data for the contiguous U.S. starting with data up to mid-April 2020, increasing in five increments of 15 days (Fig 3a and 3b) and averaging the results for the last two weeks of each study period. We found initial values of *R*(*t*_*c*_) that were large (mean/median of 2.46/2.33), started to decrease only in May and approached one in June (Table B in S1 Text). We then defined pandemic time *t*_*p*_ and estimated *R*(*t*_*p*_) the reproduction number as a function of pandemic time (a time shift for each population, Fig 3b and 3d). Although patterns of *R* in calendar and pandemic time were similar, it dropped more quickly in pandemic time and had a lower variance (Table C in S1 Text). The difference between *R* in pandemic time and calendar time was also apparent when we fitted an exponential decay model to both sets of estimates: the model was a much better explanation for the temporal pattern of *R* in pandemic time than it was for calendar time (Tables D and E in S1 Text). The daily number of deaths per capita remained far more stable when viewed in both calendar and pandemic time (Fig 3 upper panels). A histogram and heat map representation of the pandemic time evolution of *R* for the contiguous U.S. is presented in Fig F in S1 Text.

We repeated our analyses for populations outside the U.S. (Fig C of S1 Text). Here, too, we observed rich dynamics in the timeseries of reported deaths on all continents, with all profile types present that were observed in the contiguous U.S. (see above). In the Americas, the spring single peak profile was observed only in Canada and Ecuador whereas most states in Central and South America showed either the summer or (late summer peak) single peak. The two countries with a large number of deaths in South America (Brazil and Mexico) also showed a large wide peak that extended over more than three months. The data for Asia-Pacific showed an increase in death in most places only in June (e.g., the single summer peak profile). However, Iran was a notable exception, showing the spring-summer double peak profile (with the first peak having already occurred in April) followed by a third resurgence in September. In Australia (and particularly the state of Victoria) we observed the single summer peak profile (with the peak in death occurring in August/early September). Examples for the summer single peak profile were Bangladesh and Saudi Arabia, which largely avoided any excess death during the spring. The data for Europe showed clear regional grouping with Italy and Spain leading the spring single peak followed closely by most of the larger European countries.

**Fig 4:**
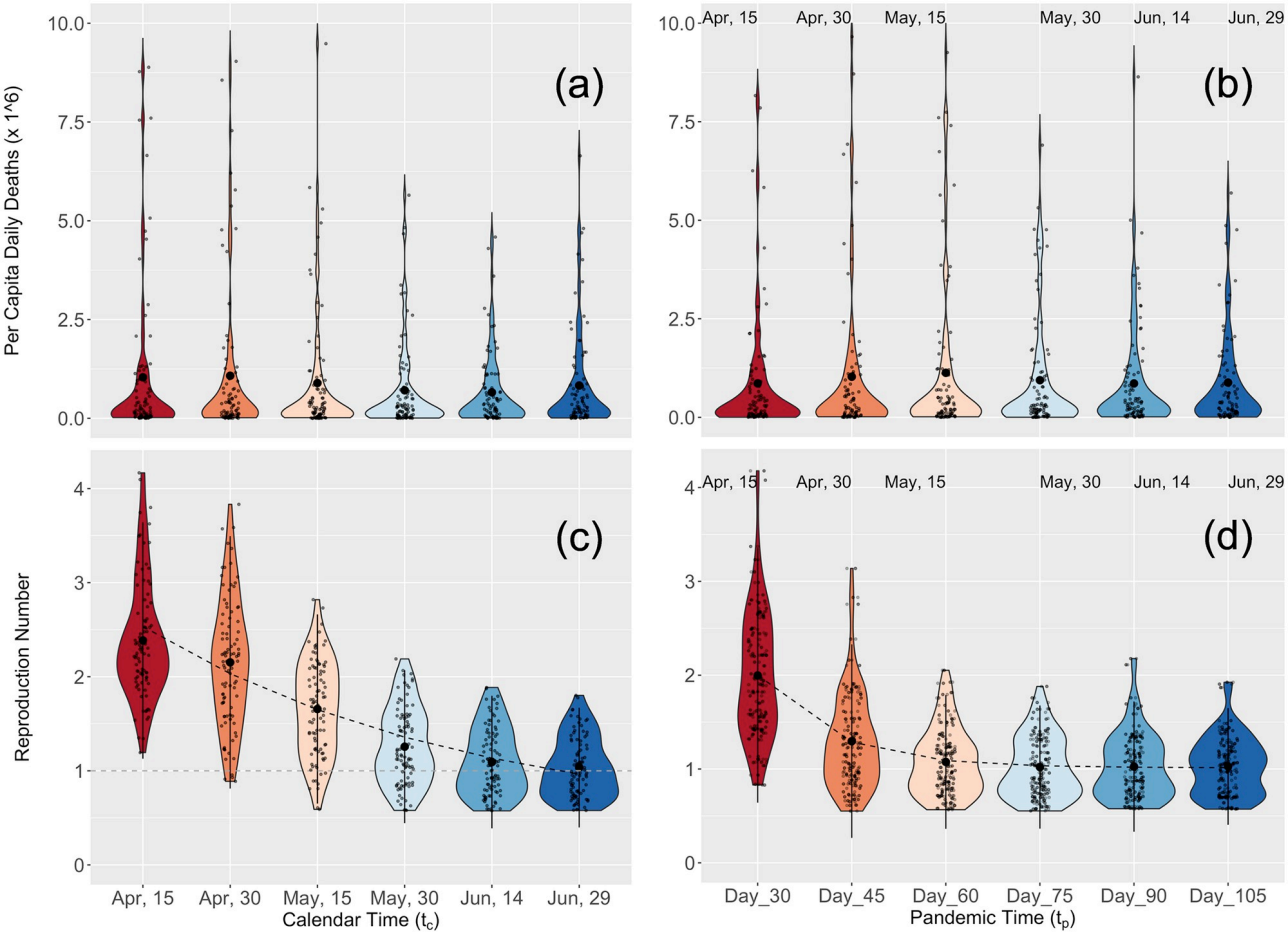
Calendar and pandemic time analysis for the world. Same as Fig 3 but for 89 world locations. (See Tables H and I of S1 Text for the exponential fit parameters).

For both calendar and pandemic time we found similar trends to those observed in the contiguous U.S. (Fig 4 and Tables F and G of S1 Text). For the calendar time reproduction number *R*(*t*_*c*_), the initial values in mid-April were above two and had a large variance (mean/median and standard deviation of 2.48/2.30 and 0.76). As with the U.S. populations, the decline in magnitude and variance of the calendar time reproduction number *R*(*t*_*c*_) was slower than the exponential-like decline of pandemic time *R*(*t*_*p*_), which hovered around a value of one for many days. However, the rate of decline in transmissibility in pandemic time was slower than that for the contiguous U.S. (compare the lower right panels of Figs 3 and 4; also Tables C, F, E and I of S1 Text). We have verified that our conclusions for the calendar and pandemic *R*(*t*) did not qualitatively change when more global locations were included in the analysis (Fig D in S1 Text). As for the contiguous U.S., here too we found that the daily number of deaths per capita remained far more stable than transmissibility when viewed in both calendar and pandemic times (Fig 4 upper panels). A histogram and heat map representation of the pandemic time evolution of *R*(*t*) is presented in Fig G in S1 Text of the Supporting Information.

For completeness, we used the Cori method [42] as implemented in the “EpiEstim” package [43] to infer the effective reproduction number for the U.S. and the world Fig H in S1 Text. We applied the method (see Appendix B in S1 Text) to the inferred daily deaths for the same calendar time window and found similar trends: initially high values that decreased and hovered around one for many days. However, the details of the time evolution were different, which is to be expected. Whereas the estimate of the effective reproduction number includes in it both the depletion of the susceptible population and the possible change in transmission, i.e., *R*_*effective*_(*t*) = *R*_0_(*t*)*S*(*t*)/*N*, our mechanistic model separately tracked the transmission term and the susceptible population and analyzed only the change in transmission.

## Discussion

We used an age-stratified compartmental model fit to different populations in the U.S. and around the world. We did not explicitly consider individual interventions as explanations for reduced values of time-dependent transmission, but rather investigated overall trends in *R*(*t*). We found a common pattern of a reduction in transmissibility during the initial period, rather than constant transmissibility, of which there were no obvious examples. Using the concept of local pandemic time, we showed that the initial value of *R*(*t*_*p*_) was ≈2 with a large variance (0.28/0.46 for the U.S./world). As the pandemic progressed, the magnitude and variance of *R*(*t*_*p*_) decreased monotonically, eventually hovering around one for a prolonged period. While the magnitude and variance of *R*(*t*_*p*_) estimated from deaths decreased consistently across the globe, the daily number of deaths themselves did not. In contrast, as a function of calendar time, the initial value of *R*(*t*_*c*_) was even larger (a mean value of 2.47 and a variance of 0.37/0.58 for the U.S./world) and the decrease in magnitude was slower, with a lower reduction in the variance.

Our study relied on a number of potentially important assumptions, approximations and limitations. First, we chose the daily deaths as the dataset to fit to the model, inferring it from cumulative confirmed deaths, as opposed to using other data such as cases, which have been associated with known and considerably larger biases. However, while the deaths are likely a more reliable measure of the pandemic than cases they are far from perfect. Different states and countries use different criteria when registering deaths (for example, some report only confirmed deaths while others report both probable and confirmed deaths) and have different delays in reporting. Additionally, the reported numbers have been shown to be lower than the true toll of the pandemic (see e.g [33, 34, 44–47]).

Our age-stratified compartmental model treated each population independently and ignored travel and importation of cases from other locations. While both global and local travel were significantly disrupted in 2020, they were responsible for the initial spread of COVID-19, and continued to play a role during latter periods. In this work, we ignored the initial seeding of cases to a location and focused on the dynamics of the virus following its introduction to a population. Also, we assumed the same quality of care over time at all locations. In practical terms, in the model, we assumed that the probability of death from COVID-19 depends only on age during this period prior to widespread vaccination. In reality, treatments for severe patients have improved over time, and they varied from one location to another [48, 49]. (We note however, that the first treatment for COVID-19, the antiviral Remdesivir, was fully approved by the FDA only on October 22, 2020 [50], which coincides with the end of the time frame of this study, and likely had little impact in most places).

A large number of studies have used available databases of interventions [8–10], coupled with statistical methods, to estimate the impact of different interventions on the effective reproduction number [15–27, 51–54]. Our approach is different from these studies in three major aspects: (i) we use a mechanistic and not a statistical model, (ii) we do not try to model or explain the impact of specific interventions, and (iii) our compartmental model estimates the change in both the susceptible population and the transmission whereas effective reproduction number based studies only estimate the product of the two, i.e. *R*_*effective*_ = *R*_0_(*t*)*S(*t*)/*N*. Because our mechanistic model estimates the depletion of suscpetibles, we are able to compare the transmissibility at different (either calendar or pandemic) time points.

The use of compartmental models with a time-varying reproduction number has been more limited. In the initial phase of the pandemic, Linka et al. [51] used a two-value time-varying reproduction number and an SEIR model to study the correlation between the reproduction number of COVID-19 and public health interventions in Europe. In another study Dickman [52] developed a deterministic SEIR model without age or spatial structure but with a three piecewise value time-dependent transmission term. Anderson et al. [53] studied the effect of social distancing measures using early (March-April 2020) case-count data from British Columbia and five other jurisdictions and an SEIR model with fractional reduction to the force of infection due to increased social distancing. Duque et al. [54] described an age and risk stratified SEIR-style model with a transmission parameter that was reduced during stay-at-home/work-safe-order time periods. Implicit in all of these studies is the assumption that interventions are similar between different populations and that they can be correlated to changes in *R*(*t*). The two major differences between our approach and these studies are: (i) we did not attempt to correlate changes in *R*(*t*) with any specific NPIs and, (ii) we used an age-stratified compartmental model with a flexible, smoothly varying, time-dependent transmission term.

Our study focused on the first wave of the COVID-19 pandemic. The monotonic reduction in the time-varying reproduction number persisted in the U.S. and globally for many days but, during the last three to four months of 2020, it began to increase in nearly all locations in the northern hemisphere, as well as many in the southern hemisphere (e.g. South Africa). The following waves were dominated by the spread of novel variants (Alpha, Beta, Delta and Omicron [7, 55, 56]). The new variants, and in particular Omicron, spread faster than the variants dominant during the time frame of this study [3–5]. To describe the emergence, and rise to dominance of a new variant, compartmental models will need to be extended to include at least two strains with different transmissibility. These subsequent waves resulted in a massive increase in cases, hospitalization and deaths that eclipsed that of the first wave, in spite of a global vaccinations effort and the development of multiple new treatments (e.g. monoclonal antibody treatments).

The general properties of the initial wave of the COVID-19 pandemic will likely remain a topic of considerable interest for many years [57]. In future studies, we plan to extend the framework developed here. Should other datasets (e.g., case counts and/or hospitalizations) become less biased and more available, we will incorporate them into the objective function we fit. Our model can also be made more flexible by including differences in quality of healthcare over time and location [48, 49], and allowing for coupling between geographic regions [29]. Our previous work on forecasting ILI in the U.S. [28–30] highlights the important role that spatial coupling can play in respiratory disease transmission, and which we anticipate will become increasingly more important as travel restrictions have been relaxed and movement between states, countries, and continents significantly increases.

In conclusion, we have characterized the observed epidemic patterns during the first months of the COVID-19 pandemic. Using an age-stratified mechanistic model with a time-dependent reproduction number and the concept of “local pandemic time” we showed a consistent pattern on four continents of an initial large magnitude and variance in the reproduction number that monotonically decreased and hovered around one for many days. We suggest that this decrease was due to a combination of specific intervention measures and changes in behaviour due to the perceived threat of COVID-19. We propose that initial planning for future respiratory pandemics should not be based on assumptions of prolonged constant transmissibility driving a rapid peak and the development of population immunity.

## Supporting information

S1 Text Appendix A: Model and Fits.
Appendix B: Effective Reproduction Number.
**Fig A: Model Sketch.** A sketch of our susceptible, exposed, infectious, recovered or dead (X) model. Each compartment is divided into 9 decadal age groups and the infectious compartments are further divided into two “holding compartments”. The rates in our model (*β*(*t*), *σ*, *γ*, *μ*, *ν*) are age independent wheres the probabilities of entering each compartment (*p*_*M*_, *p*_*F*_, *p*_*A*_, *p*_*S*_) depend strongly on age (see Table A).
**Fig B: Simulate and Recover.** Sample fits to synthetic data generated using known R(t) profiles. The synthetic daily death (red circles and left y-axis) is generated using known synthetic *R*(*t*) profiles (red line and right y-axis). The median result of the fit is shown in blue and the shaded light-blue is the 95% CI. The recovered, median, *R*(*t*) is also shown in blue along with the 95% CI in light-grey.
**Fig C: Global Inferred per capita daily death.** Inferred per capita daily reported deaths for 120 locations (other than the U.S). grouped by continent (with Australia grouped with Asia). To balance the number of locations on each continent, here we show more locations than used for the analysis of *R*. (See text for more detail). Within each panel locations are ordered by decreasing latitude from top to bottom. For each location, the date of the first reported death is marked with a black dot. For clarity, data is shown on a log scale and saturated at 60 for all panels other than the bottom right.
**Fig D: R values sensitivity analysis.** Median/mean (top/bottom row) calendar/pandemic (left/right column) R values and their standard deviations inferred using different selection procedures for 89–110 global locations. Limited: for both calendar and pandemic analysis use the same limited subset of 89 locations for which there was two weeks or more of daily inferred death data for the first calendar time calculation. Increase: use the same subset of locations for the six calendar and pandemic (R(*t*_*c*_) and R(*t*_*p*_)) analysis, but allow the number of locations to gradually increase from 89 to 110 as more locations have sufficient calendar data. Different: the calendar analysis includes the subset of 89 locations that had sufficient data at the time of the first calendar calculation and the pandemic analysis includes all 110 locations that have sufficient data for at all six pandemic times.
**Fig E: Daily inferred death fits.** Sample fits to inferred daily reported deaths (red circles and right y-axis) from 15 countries. The grey traces are 100 samples from the posterior distribution of the fit and the orange crosses denote the reported per capita cumulative deaths (no y-axis). The median and 95% confidence interval for *R*(*t*) is shown in dark and light blue with the left y-axis. Locations are ordered by decreasing cumulative deaths.
**Fig F: Time evolution of *R*(*t*_*p*_) for the U.S.** Left column: histogram plots of the pandemic time R(*t*_*p*_) values for the contiguous U.S. as calculated by the model by fitting the inferred daily reported deaths using 30, 45, 60, 75, 90 and 105 days (panels (a) to (f)) since the first reported death in each location. The black vertical dashed line is at *R*(*t*_*p*_) = 1. Right panel: a heat map representation of the data showing the value for each of the 49 contiguous jurisdictions. The map base layer was made with Natural Earth, a free vector and raster map data [9].
**Fig G: Global time evolution of *R*(*t*_*p*_).** Same as Fig F but for 110 world locations. For clarity the entire U.S. is treated as a single country in these maps and we display results for more locations than the 89 discussed in the text and tables. The map base layer was made Natural Earth, a free vector and raster map data [9].
**Fig H: Effective reproduction number analysis.** Estimated effective reproduction number for the U.S. and the world (panels (a) and (b) respectively). See Appendix Bfor details on the calculation. Whereas the overall shape of the time-dependence is similar to what we found with our time-dependent reproduction number (see Figs 3 and 4) the details are not. See text for more details.
**Table A: Severity Probabilities.** Age dependent probabilities of entering the mild, flu-like, asymptomatic or sever infectious compartments (*p*_*M*_, *p*_*F*_, *p*_*A*_, *p*_*S*_, respectively) based on [1].
**Table B: Calendar time reproduction number.** Estimated R(*t*_*c*_) values for the contiguous U.S. (49 jurisdiction) as a function of calendar time. Numbers in parentheses denote the 95% confidence interval of the mean and median values and SD denotes standard deviation. For each jurisdiction and study period (i.e. Date), the start date of the calculation is the date of first reported death. The reported apparent reproduction number was calculated as the average value of *R*(*t*_*c*_) over the last two weeks of each study period.
**Table C: Pandemic time reproduction number.** Same as Table B but as a function of local pandemic time R(*t*_*p*_). For each jurisdiction, each study period includes 30, 45, .., 105 days since the first reported death. The reported apparent reproduction number was calculated as the average value of *R*(*t*_*p*_) over the last two weeks of the local pandemic time.
**Table D: Exponential Fits to R.** Results of exponential fit *R*(*t*) = *R*_∞_ + (*R*_0_ − *R*_∞_)*e*^−*αt*^ to calendar R(*t*_*c*_) values for the 49 U.S. jurisdictions.
**Table E: Exponential Fits to R.** Same as Table D but for pandemic R(*t*_*p*_) values for the 49 U.S. jurisdictions.
**Table F: Calendar time reproduction number.** Estimated R(*t*_*c*_) values for 89 global locations as a function of calendar time. Numbers in parentheses denote the 95% confidence interval of the mean and median values and SD denotes standard deviation.
**Table G: Pandemic time reproduction number.** Same as Table F but as a function of local pandemic time, R(*t*_*p*_).
**Table H: Exponential Fits to R.** Results of exponential fit *R*(*t*) = *R*_∞_ + (*R*_0_ − *R*_∞_)*e*^−*αt*^ to calendar R(*t*_*c*_) values for the 89 global locations.
**Table I: Exponential Fits to R.** Same as Table H but for pandemic R(*t*_*p*_) values for the 89 global locations.
(PDF)
