# Supplementary material for: Consistent pattern of epidemic slowing across many geographies led to longer, flatter initial waves of the COVID-19 pandemic
Source: PLoS Comput Biol. 2022 Aug 15;18(8):e1010375. doi: 10.1371/journal.pcbi.1010375 (PMC9410547; doi:10.1371/journal.pcbi.1010375)
Supplement: S1 Text — Appendix A: Model and Fits. Appendix B: Effective Reproduction Number. Fig A: Model Sketch. A sketch of our susceptible, exposed, infectious, recovered or dead (X) model. Each compartment is divided into 9 decadal age groups and the infectious compartments are further divided into two “holding compartments”. The rates in our model (β(t), σ, γ, μ, ν) are age independent wheres the probabilities of entering each compartment (pM, pF, pA, pS) depend strongly on age (see Table A). Fig B: Simulate and Recover. Sample fits to synthetic data generated using known R(t) profiles. The synthetic daily death (red circles and left y-axis) is generated using known synthetic R(t) profiles (red line and right y-axis). The median result of the fit is shown in blue and the shaded light-blue is the 95% CI. The recovered, median, R(t) is also shown in blue along with the 95% CI in light-grey. Fig C: Global Inferred per capita daily death. Inferred per capita daily reported deaths for 120 locations (other than the U.S). grouped by continent (with Australia grouped with Asia). To balance the number of locations on each continent, here we show more locations than used for the analysis of R. (See text for more detail). Within each panel locations are ordered by decreasing latitude from top to bottom. For each location, the date of the first reported death is marked with a black dot. For clarity, data is shown on a log scale and saturated at 60 for all panels other than the bottom right. Fig D: R values sensitivity analysis. Median/mean (top/bottom row) calendar/pandemic (left/right column) R values and their standard deviations inferred using different selection procedures for 89–110 global locations. Limited: for both calendar and pandemic analysis use the same limited subset of 89 locations for which there was two weeks or more of daily inferred death data for the first calendar time calculation. Increase: use the same subset of locations for the six calendar and pandemic (R(tc) and R(tp) [file pcbi.1010375.s001.pdf]

Supporting information

Appendix A. Model and Fits Our age structured model includes susceptible (S), exposed (E), infectious (I), recovered (R), hospitalized (H) and deceased (X) compartments and is illustrated in Fig A. Each of these compartments is divided into 9 decadal age groups: 0-9, 10-19,..,70-79, and 80+. The infectious compartment has four severity levels: I_M is the mild compartment, I_F is the flu-like compartment, I_A is the asymptomatic compartment and I_S is the severe compartment. The age dependent probability of entering the flu-like and severer compartments (p_F and p_S) was taken from [1], and p_A is assumed to be 0.4 for all ages and p_M is calculated for each age as one minus the sum of the three other probabilities. For the purpose of this analysis only the age dependent values of p_S contribute to the fitting of the daily inferred mortality. All the probabilities are provided in Table A. The severe infectious results in hospitalization which may end in either recovery or death (with equal probabilities, i.e. $p_D = 0.5$). The severe compartment is the only path to hospitalization. To obtain the proper temporal distribution for the generation time, we divided each of the four infectious compartments into two "holding compartments" (e.g, an individual with mild symptoms first enters the holding compartment I_{1M}, from there they transition to I_{2M} and then exit to R). This, and our selection of parameters, ensures that individuals start exiting the infectious state after a minimum stay of two days with a mean time to recovery of 6 days. The time-dependent transmission term, $\beta(t)$ was assumed to be age independent as was the infectiousness (see below). A key component in the model is the age-dependent force of infection vector ($\vec{\lambda}$) which was evaluated at each time step by first calculating the total number of infectious individuals in each age group

$$\vec{I}(t) = \sum_{i=1}^{2}\sum_{j=1}^{4} \vec{I}_{ij}(t), \tag{1}$$

where the first sum is over the two "holding compartments" and the second sum is over the four levels of severity. We use an arrow to denote vectors and (below) bold letters to denote matrices. Using the number of infectious individuals in each age group, along with the infectiousness vector ($\vec{V}_{Infv}$, and the age and country specific contact matrix [2] ($\mathbf{M}$) we calculated the age-dependent force of infection vector as

$$\vec{\lambda} = \beta(t) \cdot \frac{I(\vec{t})}{N} \odot (\vec{V}_{Infv} \cdot \mathbf{M}). \tag{2}$$

Here the symbol $\odot$ denotes an element-by-element product of two vectors and N is the total population. In the limit of a single age group and a single infectious compartment this equation for the force of infection reduces to the expected $\beta(t)I(t)/N$ form. Finally, the time-dependent transmission term, $\beta(t)$, was defined using our flexible form for the transmission rate

$$\beta(t) = \frac{R(t)}{\gamma}, \tag{3}$$

where

$$R(t) = \frac{1}{2}\left[R_0 + R_N + \sum_{n=1}^{N}\left((R_n - R_{n-1})\tanh\left(\frac{t - t_{n-1}}{L}\right)\right)\right]. \tag{4}$$

As noted in the main text, this form is a generalization of our previous two-value model for $R(t)$ [3–5]. It produces a continuous curve where at roughly time t_{n-1} the value of $R(t)$ transitions smoothly (over the course of $\approx 2L$ days) from a value of R_{n-1} to R_n. In the absence of age specific infectiousness estimates, we assumed that it is age

independent and set to $1/n_{ages}$ where n_{ages} is the number of age groups in the model. We set the latent period and and total recovery time (σ and γ in Fig A to two and six days, respectively. This implies that on average individuals spend three days in each of the infectious “holding compartments”. Finally, since we made no attempt to model the delay in reporting of deaths, or to track the rate at which hospitalized individuals recover or die, we set both rates (μ and ν in Fig A) to three days.

For each location, we simulated a retrospective study by repeating the fitting procedure using an increasing number of either calendar or local pandemic days. We used an MCMC fitting procedure (with 10^6 steps) to optimize the parameters ($R_0, ..R_N$ and $t_0, .., t_{n-1}$) that determine the time-dependent transmission term, $R(t)$. (The timescale of variation was set to approximately seven days using $L = 3$ in Equation 4.) The rate parameters and the age dependent probabilities of entering the infectious compartments were kept fixed at the values described above. The objective function in the fitting procedure was a Poisson-based Log-Likelihood, and the fitting maximized the probability that the inferred daily reported death is a Poisson expression of the inferred daily incidence death. Multiple models of $R(t)$ (with 2, 3, 4 and 5 values, i.e. $N = 1$, ..., 4) were fit to each location and the AICc [6] score was calculated for each model. We selected the best N based on the AICc score (provided the effective chain size of all the parameters was greater than 50). This was repeated for each location and study period. The reported apparent reproduction number was calculated as the average value of $R(t)$ for the selected N over the last two weeks of each study period.

Appendix B. Effective Reproduction Number We used the Cori method [7] as implemented in the “EpiEstim” package [8] to estimate the effective reproduction number as a function of time. For consistency we used the same data for this fit: the inferred daily death for 49 U.S. jurisdictions and 89 global locations. We assumed that the mean and standard deviation (sd) of the serial interval (SI) distribution are not well defined and are thus drawn from a truncated Normal distribution: Normal(mean,sd). For the mean of the SI we used a Normal(6.48,3.83) truncated at 2.48 and 10.48 and for the sd we used a Normal(10, 1) truncated at 1 and 19. We used the default weekly sliding window for the estimate and applied it to the same time window as the one used for the calendar time calculations.

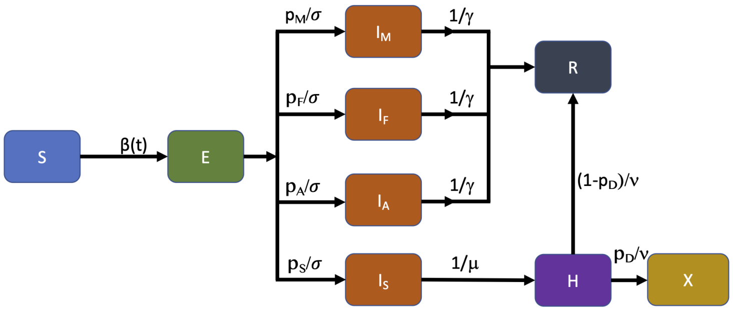

Fig A. Model Sketch A sketch of our susceptible, exposed, infectious, recovered or dead (X) model. Each compartment is divided into 9 decadal age groups and the infectious compartments are further divided into two "holding compartments". The rates in our model $(\beta(t), \sigma, \gamma, \mu, \nu)$ are age independent wheres the probabilities of entering each compartment (p_M, p_F, p_A, p_S) depend strongly on age (see Table A).

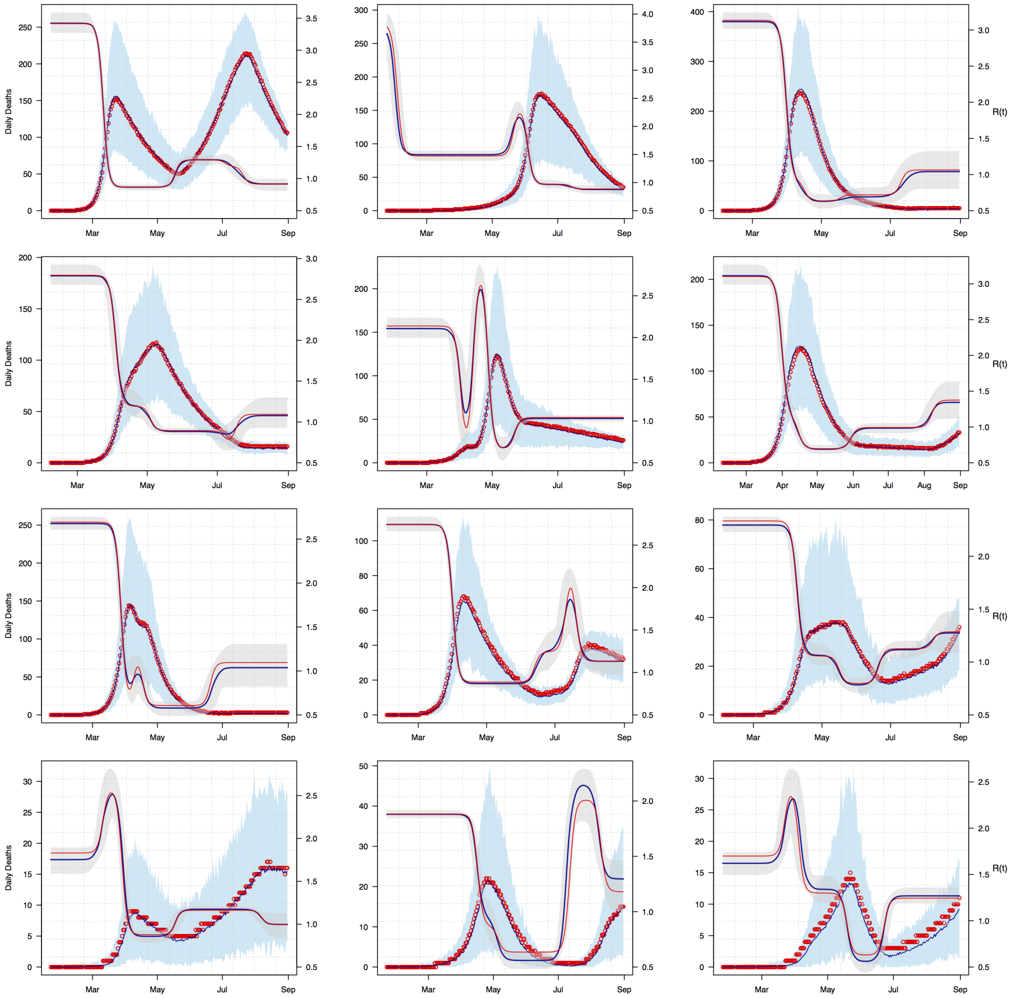

Fig B. Simulate and Recover. Sample fits to synthetic data generated using known R(t) profiles. The synthetic daily death (red circles and left y-axis) is generated using known synthetic $R(t)$ profiles (red line and right y-axis). The median result of the fit is shown in blue and the shaded light-blue is the 95% CI. The recovered, median, $R(t)$ is also shown in blue along with the 95% CI in light-grey.

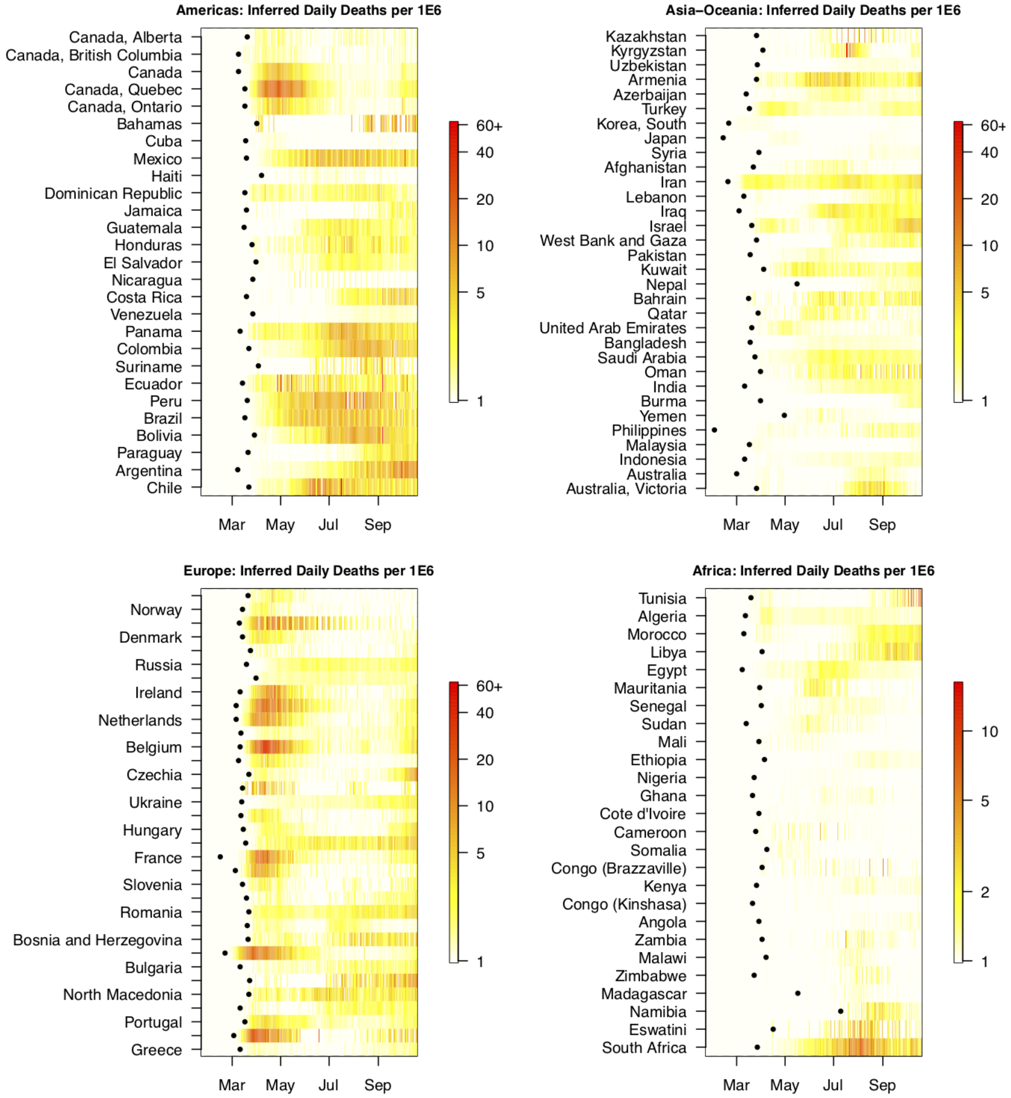

Fig C. Global Inferred per capita daily death. Inferred per capita daily reported deaths for 120 locations (other than the U.S.) grouped by continent (with Australia grouped with Asia). To balance the number of locations on each continent, here we show more locations than used for the analysis of R. (See text for more detail.) Within each panel locations are ordered by decreasing latitude from top to bottom. For each location, the date of the first reported death is marked with a black dot. For clarity, data is shown on a log scale and saturated at 60 for all panels other than the bottom right.

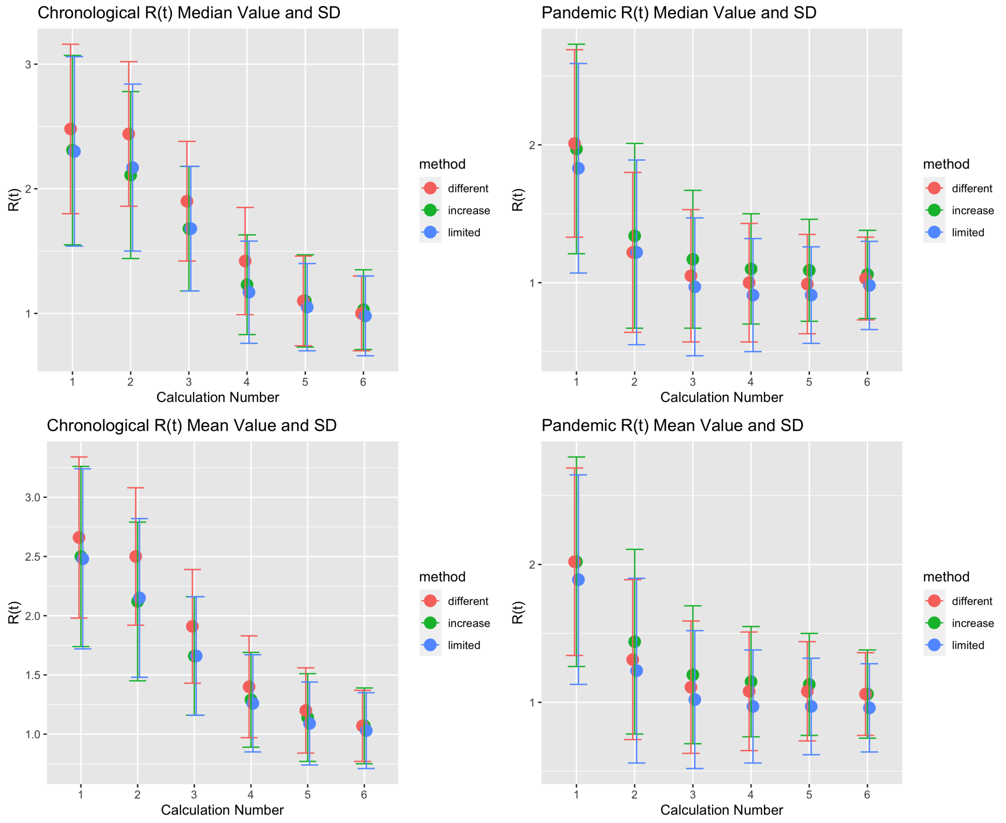

Fig D. R values sensitivity analysis. Median/mean (top/bottom row) calendar/pandemic (left/right column) R values and their standard deviations inferred using different selection procedures for 89-110 global locations. Limited: for both calendar and pandemic analysis use the same limited subset of 89 locations for which there was two weeks or more of daily inferred death data for the first calendar time calculation. Increase: use the same subset of locations for the six calendar and pandemic (R(t_c) and R(t_p)) analysis, but allow the number of locations to gradually increase from 89 to 110 as more locations have sufficient calendar data. Different: the calendar analysis includes the subset of 89 locations that had sufficient data at the time of the first calendar calculation and the pandemic analysis includes all 110 locations that have sufficient data for at all six pandemic times.

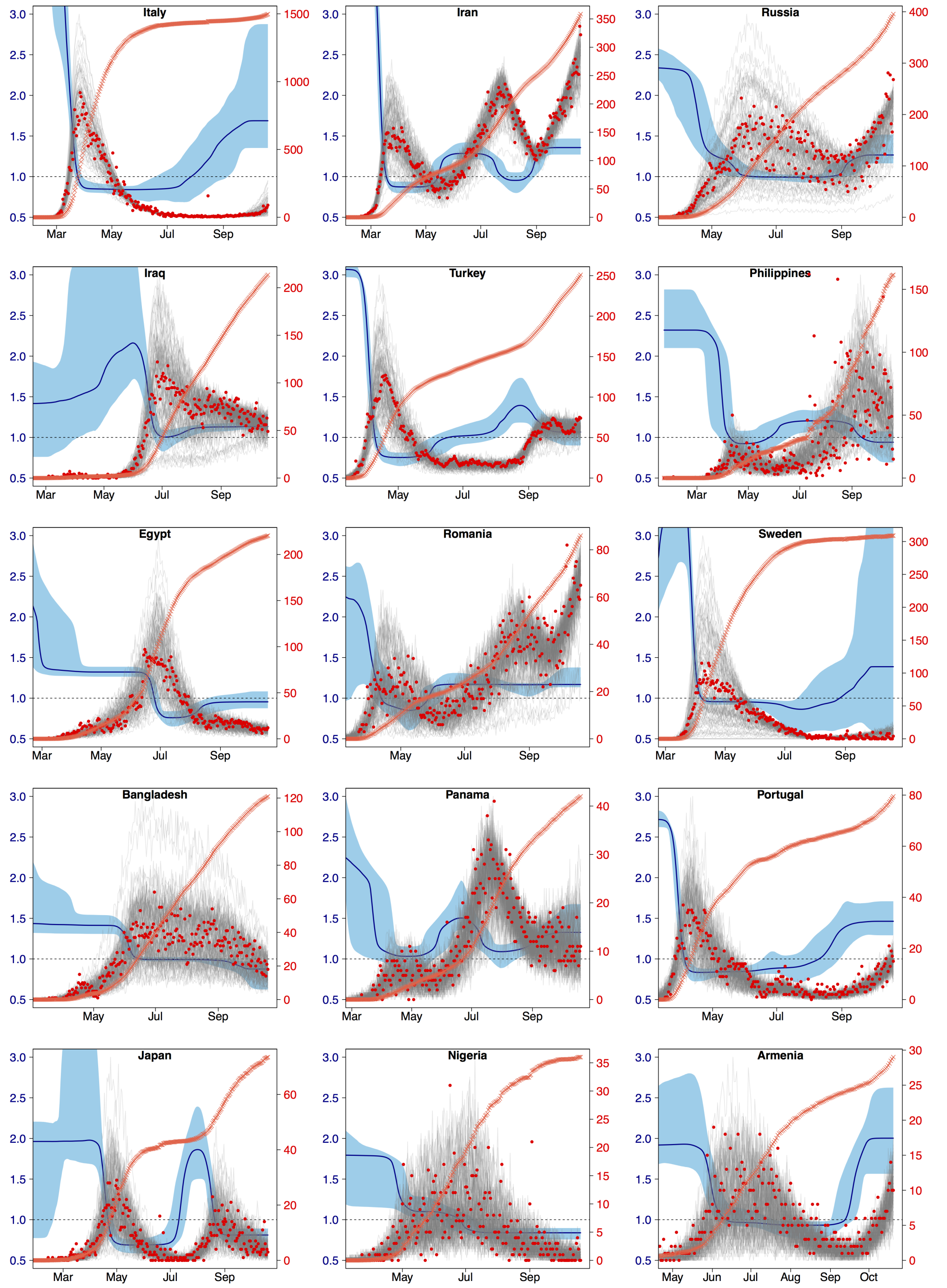

Fig E. Daily inferred death fits. Sample fits to inferred daily reported deaths (red circles and right y-axis) from 15 countries. The grey traces are 100 samples from the posterior distribution of the fit and the orange crosses denote the reported per capita cumulative deaths (no y-axis). The median and 95% confidence interval for $R(t)$ is shown in dark and light blue with the left y-axis. Locations are ordered by decreasing cumulative deaths.

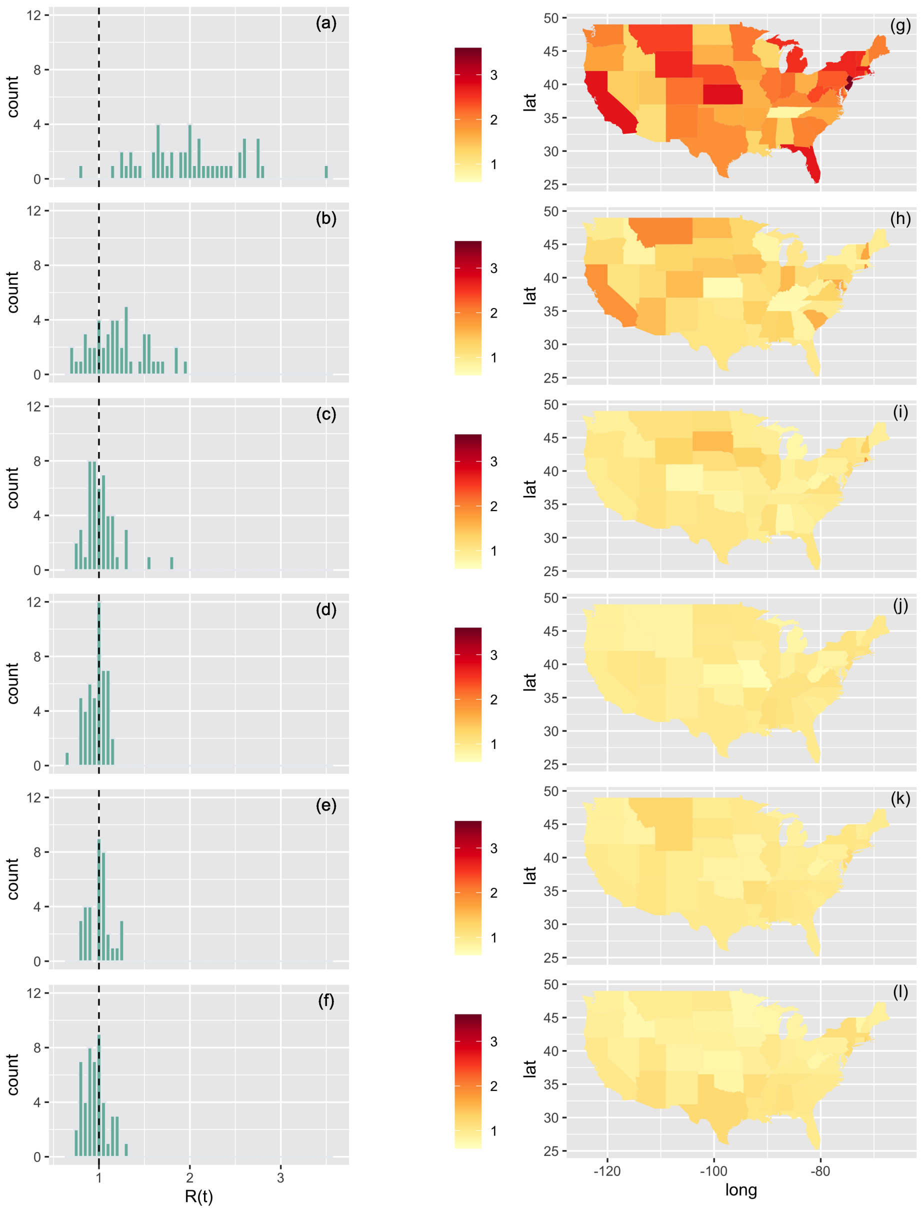

Fig F. Time evolution of $R(t_p)$ for the U.S. Left column: histogram plots of the pandemic time R(t_p) values for the contiguous U.S. as calculated by the model by fitting the inferred daily reported deaths using 30, 45, 60, 75, 90 and 105 days (panels (a) to (f)) since the first reported death in each location. The black vertical dashed line is at $R(t_p) = 1$. Right panel: a heat map representation of the data showing the value for each of the 49 contiguous jurisdictions. The map base layer was made with Natural Earth, a free vector and raster map data [9].

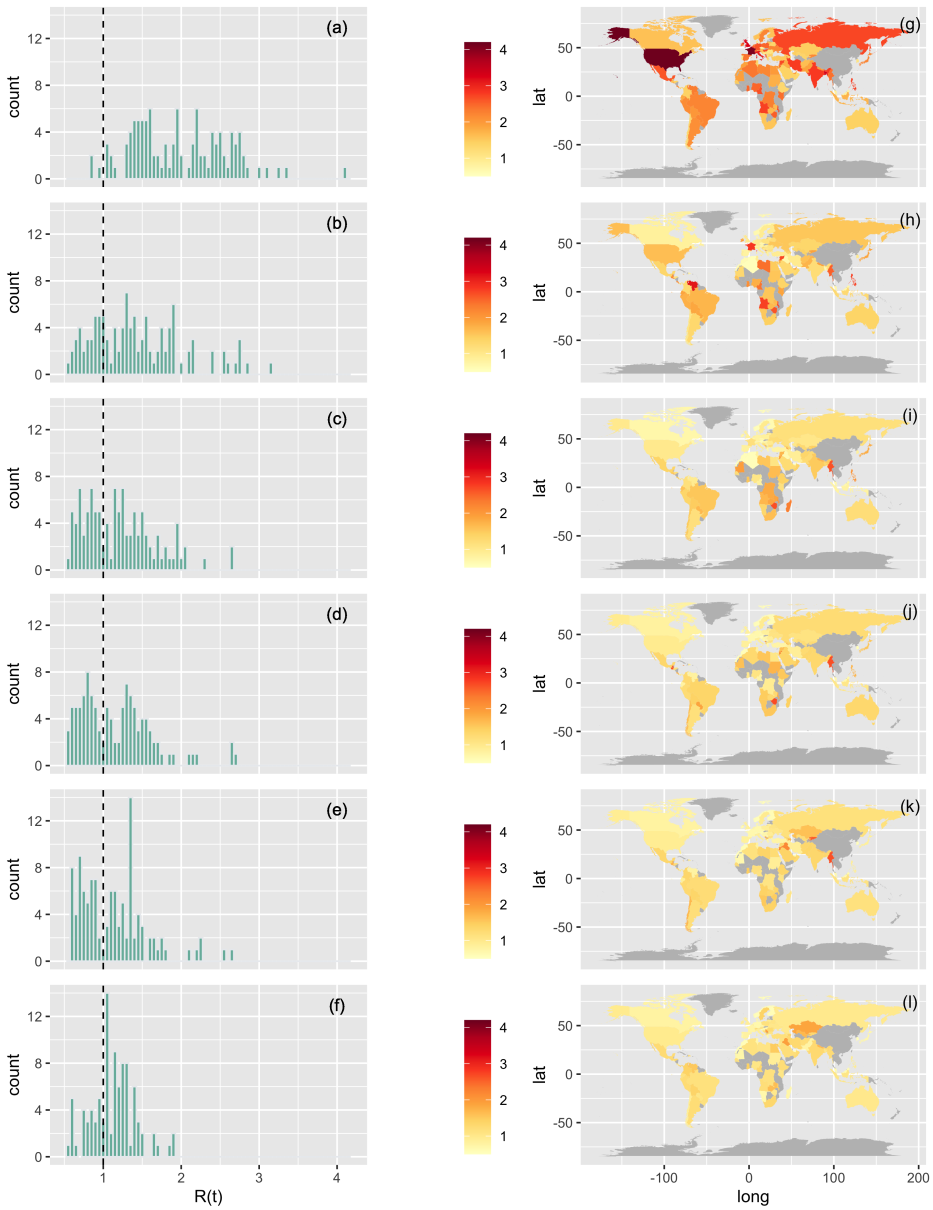

Fig G. Global time evolution of $R(t_p)$. Same as Fig F but for 110 world locations. For clarity the entire U.S. is treated as a single country in these maps and we display results for more locations than the 89 discussed in the text and tables. The map base layer was made Natural Earth, a free vector and raster map data [9].

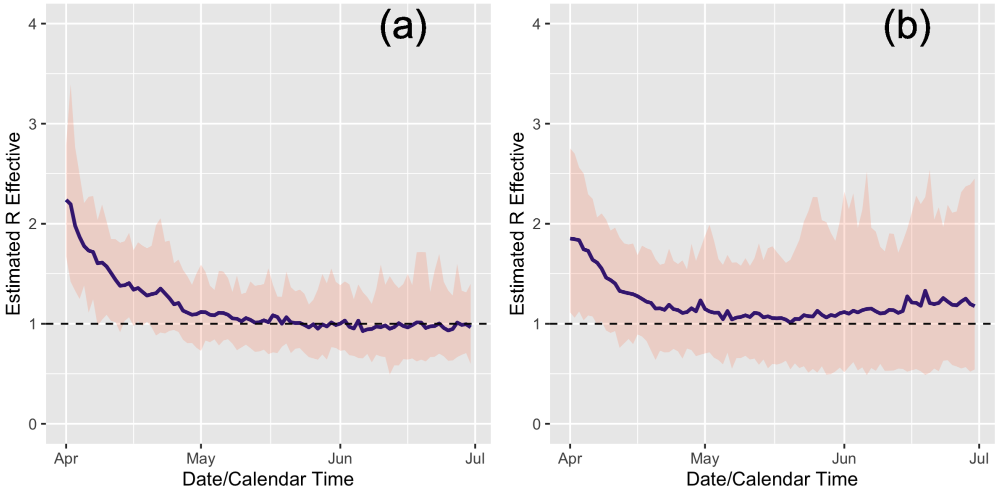

Fig H. Effective reproduction number analysis. Estimated effective reproduction number for the U.S. and the world (panels (a) and (b) respectively). See Appendix Bfor details on the calculation. Whereas the overall shape of the time-dependence is similar to what we found with our time-dependent reproduction number (see Figs 3 and 4) the details are not. See text for more details.

Table A. Severity Probabilities. Age dependent probabilities of entering the mild, flu-like, asymptomatic or sever infectious compartments (p_M, p_F, p_A, p_S, respectively) based on [1].

Age group	p_M	p_F	p_A	p_S
0-9	0.0	0.5999839	0.4	0.0000161
10-19	0.00041	0.5995205	0.4	0.0000695
20-29	0.0140	0.5856910	0.4	0.0003090
30-39	0.0343	0.5648560	0.4	0.0008440
40-49	0.0425	0.5558900	0.4	0.0016100
50-59	0.0816	0.5124500	0.4	0.0059500
60-69	0.118	0.4627000	0.4	0.0193000
70-80	0.166	0.3912000	0.4	0.0428000
80+	0.184	0.3380000	0.4	0.0780000

Table B. Calendar time reproduction number. Estimated R(t_c) values for the contiguous U.S. (49 jurisdiction) as a function of calendar time. Numbers in parentheses denote the 95% confidence interval of the mean and median values and SD denotes standard deviation. For each jurisdiction and study period (i.e. Date), the start date of the calculation is the date of first reported death. The reported apparent reproduction number was calculated as the average value of $R(t_c)$ over the last two weeks of each study period.

Date	Mean	Median	SD	#Below one
04-15-2020	2.46(2.28-2.64)	2.33(2.15-2.54)	0.61	0
04-30-2020	2.38(2.21-2.55)	2.25(2.13-2.71)	0.59	0
05-15-2020	1.98(1.87-2.10)	1.91(1.78-2.09)	0.41	0
05-30-2020	1.41(1.30-1.53)	1.32(1.15-1.58)	0.41	5
06-14-2020	1.20(1.10-1.29)	1.04(1.00-1.13)	0.34	17
06-29-2020	1.07(1.01-1.14)	1.00(0.95-1.05)	0.22	23

Table C. Pandemic time reproduction number. Same as Table B but as a function of local pandemic time R(t_p). For each jurisdiction, each study period includes $30, 45, .., 105$ days since the first reported death. The reported apparent reproduction number was calculated as the average value of $R(t_p)$ over the last two weeks of the local pandemic time.

Days since 1st death	Mean	Median	SD	#Below one
30	1.99(1.84-2.14)	2.00(1.73-2.11)	0.53	1
45	1.22(1.13-1.30)	1.19(1.08-1.28)	0.31	13
60	1.03(0.97-1.08)	1.00(0.94-1.06)	0.19	25
75	0.97(0.94-1.00)	1.00(0.94-1.02)	0.10	27
90	0.99(0.96-1.02)	0.97(0.96-1.01)	0.11	30
105	0.96(0.92-1.00)	0.94(0.90-0.99)	0.13	33

Table D. Exponential Fits to R. Results of exponential fit $R(t) = R_\infty + (R_0 - R_\infty) e^{-\alpha t}$ to calendar R(t_c) values for the 49 U.S. jurisdictions.

Parameter	Estimate	Standard Error	$Pr(> \|t\|)$
R_∞	-5.37	10.98	0.62
R_0	2.57	0.061	$< 2^{-16}$
α	0.003	$6.6 * 10^{-4}$	$1.97 * 10^{-4}$

Table E. Exponential Fits to R. Same as Table D but for pandemic R(t_p) values for the 49 U.S. jurisdictions.

Parameter	Estimate	Standard Error	$Pr(> \|t\|)$
R_∞	0.97	0.024	$< 2^{-16}$
R_0	1.99	0.039	$< 2^{-16}$
α	0.094	0.0017	$< 2^{-16}$

Table F. Calendar time reproduction number. Estimated R(t_c) values for 89 global locations as a function of calendar time. Numbers in parentheses denote the 95% confidence interval of the mean and median values and SD denotes standard deviation.

Date	Mean (95% CI)	Median (95%) CI	Standard Deviation	# Below one
04-15-2020	2.48(2.32-2.64)	2.30(2.16-2.46)	0.76	0
04-30-2020	2.15(2.02-2.29)	2.17(1.93-2.26)	0.67	4
05-15-2020	1.66(1.55-1.76)	1.68(1.51-1.83)	0.50	9
05-30-2020	1.26(1.17-1.34)	1.17(1.08-1.42)	0.41	27
06-14-2020	1.09(1.02-1.17)	1.05(0.92-1.13)	0.35	44
06-29-2020	1.03(0.97-1.10)	0.98(0.90-1.07)	0.32	49

Table G. Pandemic time reproduction number. Same as Table F but as a function of local pandemic time, R(t_p).

Days Since 1st Death	Mean (95% CI)	Median (95% CI)	SD	#Below one
30	2.00(1.85-2.14)	1.90(1.63-2.21)	0.68	3
45	1.30(1.19-1.41)	1.24(1.06-1.33)	0.52	30
60	1.08(1.00-1.15)	1.02(0.90-1.17)	0.36	43
75	1.02(0.95-1.09)	0.95(0.85-1.08)	0.33	48
90	1.03(0.95-1.10)	0.94(0.87-1.12)	0.35	47
105	1.03(0.96-1.09)	1.04(0.90-1.13)	0.31	42

Table H. Exponential Fits to R. Results of exponential fit $R(t) = R_\infty + (R_0 - R_\infty) e^{-\alpha t}$ to calendar R(t_c) values for the 89 global locations.

Parameter	Estimate	Standard Error	$Pr(> \|t\|)$
R_∞	0.42	0.26	0.117
R_0	2.55	0.052	$< 2^{-16}$
α	0.018	$4.4 * 10^{-4}$	$< 2^{-16}$

Table I. Exponential Fits to R. Same as Table H but for pandemic R(t_p) values for the 89 global locations.

Parameter	Estimate	Standard Error	$Pr(>\|t\|)$
R_∞	1.01	0.030	$< 2^{-16}$
R_0	2.00	0.047	$< 2^{-16}$
α	0.094	0.0014.	$< 2^{-16}$

References

1. Verity R, Okell LC, Dorigatti I, Winskill P, Whittaker C, Imai N, et al. Estimates of the severity of coronavirus disease 2019: a model-based analysis. Lancet Infect Dis. 2020;20(6):669–677.

2. Walker PGT, Whittaker C, Watson OJ, Baguelin M, Winskill P, Hamlet A, et al. The impact of COVID-19 and strategies for mitigation and suppression in low- and middle-income countries. Science. 2020;369(6502):413–422.

3. Riley P, Ben-Nun M, Linker JA, Cost AA, Sanchez JL, George D, et al. Early Characterization of the Severity and Transmissibility of Pandemic Influenza Using Clinical Episode Data from Multiple Populations. PLoS Comput Biol. 2015;11(9):e1004392.

4. Ben-Nun M, Riley P, Turtle J, Bacon DP, Riley S. Forecasting national and regional influenza-like illness for the USA. PLoS Comput Biol. 2019;15(5):e1007013.

5. Turtle J, Riley P, Ben-Nun M, Riley S. Accurate influenza forecasts using type-specific incidence data for small geographical units. PLoS Comput Biol. 2021; p. e1009230.

6. Akaike H. A new look at the statistical model identification. IEEE Trans Automat Contr. 1974;19(6):716–723.

7. Cori A, Ferguson NM, Fraser C, Cauchemez S. A New Framework and Software to Estimate Time-Varying Reproduction Numbers During Epidemics. Am J Epidemiol. 2013;178(9):1505–1512.

8. Cori A, Kamvar Z, Stockwin J, Jombart T, Dahlqwist E, FitzJohn R, et al.. EpiEstim v2.2-3: A tool to estimate time varying instantaneous reproduction number during epidemics; 2021. https://github.com/mrc-ide/EpiEstim.

9. Natural Earth, Free vector and raster map data;. https://www.naturalearthdata.com/.
